# The Impact of AI Guide Language Strategies on Museum Visitor Experience: The Mediating Role of Psychological Distance in the Arousal–Topic Fit Effect

**DOI:** 10.3390/bs15111569

**Published:** 2025-11-17

**Authors:** Tiansheng Xia, Yujiao Wu, Aopeng Qiu, Ziyu Liu, Meng Fan

**Affiliations:** 1 School of Art & Design, Guangdong University of Technology, Guangzhou 510090, China; 2 Mental Health Education Center, Guangdong University of Technology, Guangzhou 510006, China

**Keywords:** AI digital guide, language awareness, psychological distance, emotional resonance, museum experience

## Abstract

AI digital human tour guides are increasingly used in museums, yet current designs prioritize technological features over content adaptability, neglecting the psychological underpinnings of effective communication. This study examines how language arousal (high vs. low) influences visitor satisfaction, continued use intention, and knowledge recall, with a focus on the mediating roles of psychological distance, emotional resonance, and concentration. The experiment employed a 2 × 2 mixed design, with language arousal as the between-subjects variable and science popularization themes as the within-subjects variable. Eighty participants were randomly assigned to either a high-arousal or low-arousal group. All participants received stimuli from both natural science and social culture science popularization themes, with presentation order balanced using the ABBA method. Results revealed a significant interaction between language arousal and topic: high-arousal language improved outcomes in natural science contexts by reducing psychological distance, whereas low-arousal language was more effective for social history, where increased psychological distance preserved perceived seriousness and credibility. Psychological distance emerged as a key mediator, while emotional resonance and concentration did not show significant effects. These findings reveal how topic-aware language design can enhance AI-guided museum experiences. The study helps AI explanation systems achieve a qualitative transformation from “being able to speak” to “being effective in speaking”.

## 1. Introduction

In recent years, the rapid evolution of artificial intelligence (AI) technology has irreversibly reshaped the ecological landscape of cultural communication. From the panoramic presentation of virtual exhibitions to the personalized services of smart guided tours, technology empowers cultural institutions to transcend physical boundaries and temporal constraints, enabling remote access to collections and personalized touring beyond traditional opening hours (Choi & Kim, 2023). Museums, as important carriers of human civilization memory (O’Byrne et al., 2018), are undergoing an intelligent transformation that has become a core issue in the development of the industry (Jiang et al., 2022). In this process, digital human guides leverage their human-like interaction capabilities (Chen et al., 2024; Y. Wang & Zhou, 2025). Bolstered by unique advantages such as 24-h service and multi-scenario adaptation, they have been upgraded from an auxiliary tool to a core medium connecting audiences and exhibits (Kefi et al., 2024; Stelmaszczyk et al., 2025). They not only accurately output cultural relic information but also, through expression simulation, motion response (Kum & Lee, 2022), and other multimodal interactions, create a more immersive visitor experience (Sung et al., 2022), thereby becoming a key factor in enhancing the public’s cultural experience (Y. Wang & Zhou, 2025).

However, current research on digital human guides remains entrenched in the “technology-first” paradigm (Tastemirova et al., 2022), excessively focusing on functional metrics such as visual authenticity and vocal fluency (Loveys et al., 2020). This purely technical approach suffers from a fundamental limitation: it overlooks the psychological adaptation required for digital humans as carriers of emotion and cognition (Perlovsky, 2013). Cognitive psychology research demonstrates that effective communication must align with the audience’s information processing patterns (Trémolière et al., 2016), while there exists an inherent gap between technological implementation and psychological needs (De Araujo & Aquino Junior, 2014). The widespread application of standardized language templates (Palombini, 2017) directly reflects this research bias. When identical expression paradigms are applied to both bronze ware and modern art, or when the same narrative logic is used for both children and professional scholars, the fundamental problem lies in neglecting the cognitive characteristics and emotional needs of different audience groups (Lindquist, 2021). Museum learning theory indicates that effective cultural communication requires establishing cognitive bridges between content and visitors’ prior knowledge, a necessity that standardized language strategies cannot fulfill. This adaptation deficiency reduces digital humans to “talking information boards” (Escalas & Stern, 2003), severely undermining their value in cultural communication. The reason multisensory experiences have become an important direction for museum transformation (Pietroni, 2025) is precisely their ability to deepen memory encoding and emotional experience by enhancing situational immersion (Magalhães et al., 2025), effectively compensating for the emotional connection deficiency inherent in purely technical approaches.

Research in consumer behavior has established that language arousal is a critical variable affecting information reception (Cascio Rizzo et al., 2024). By modulating emotional intensity and information density, it activates the limbic system (Fu et al., 2020), thereby influencing audience attention and memory formation (Berger et al., 2023). However, the application of this theory in museum contexts faces three significant limitations: First, existing research has failed to establish a matching model between language arousal levels and popular science topic types. Historical narratives rely on scene reconstruction and emotional resonance (Shi & Tian, 2025), while scientific explanations emphasize logical reasoning (Dreyer & Van Der Walt, 2023). Current standardized language strategies struggle to accommodate these distinct cognitive demands, often resulting in content that is either overly sentimental or excessively dry. Second, visitors’ cultural backgrounds, knowledge levels, and other factors influence their psychological distance from exhibits (Altweck & Marshall, 2015; Lobban & Murphy, 2020). When digital guides use language that mismatches the audience’s cognitive framework—such as employing professional terminology with children—it not only fails to reduce psychological distance but may even exacerbate cognitive barriers (Nook et al., 2017). Existing research has yet to systematically elucidate how language strategies dynamically regulate psychological distance. Third, digital human guides remain largely confined to information transmission (Q. Liu et al., 2023) and have failed to achieve the transition from “functional service” to “emotional connection” through rhythmic language modulation and narrative design (Palombini, 2017). This represents a significant gap compared to the immersive experience goals pursued in museum intelligentization (Sung et al., 2022).

Based on these limitations, this study proposes the following core research question: How can topic-based language arousal strategies be developed to optimize the communicative efficacy of digital guides by regulating psychological distance and constructing immersive experiences? By integrating Psychological Distance Theory (Massara & Severino, 2013) and Language Expectancy Theory (B. Wang et al., 2025), we aim to explore how language design can alleviate psychological barriers between audiences, exhibits, and culture, thereby advancing the evolution of digital guides from “technological products” to “cultural media”.

This paper is structured as follows: Section 2 develops hypotheses based on Language Expectancy Theory and Psychological Distance Theory; Section 3 details the 2 × 2 mixed experimental design and measurements; Section 4 reports ANOVA and moderated mediation results; Section 5 discusses theoretical and practical implications; Section 6 concludes with limitations and future directions.

## 2. Literature Review

### 2.1. Language Expectancy Theory

Language Expectancy Theory (LET) was proposed by Michael Burgoon in 1970. It is based on persuasion theory in language and psychology (Burgoon et al., 2002). This theory assumes that language is a rule-based system, and that people have expected norms for appropriate language use in specific contexts, while unexpected language use can influence the recipient’s attitude towards persuasive messages. According to language expectancy theory, individuals form expectations about appropriate language use based on social norms, situational context, and speaker characteristics (Burgoon et al., 2002). These expectations influence their acceptance or rejection of linguistic information (Burgoon et al., 2002). High-arousal language typically contains strong emotional vocabulary, vivid rhetoric, and a compact information structure, effectively activating the brain’s limbic system (Berger et al., 2023). In contrast, low-arousal language emphasizes logic and objective presentation. It is noteworthy that language expectancy is significantly context-dependent—audiences may have different expectations for explanations of different subject matter (e.g., social history versus natural sciences). This difference is particularly evident in museum digital human explanations. B. Wang et al. (2025) propose that virtual influencers (digital humans) should use a high-arousal language style that aligns with consumers’ language expectations. This is because their energy, enthusiasm, and excitement complement their personas, creating an essential dramatic effect. Cascio Rizzo et al. (2024) further point out that the impact of high-arousal language on micro-influencers and macro-influencers is inconsistent. Language expectancy theory provides an important theoretical framework for the language design of digital human tour guides. This theory emphasizes that audiences have specific expectations of the communicator’s language expression, and when actual language characteristics match these expectations, a more positive effect is produced (B. Wang et al., 2025). In museum settings, language arousal, as a key variable, can influence audience cognitive processing through emotional intensity, rhythmic tension, and information density (Cascio Rizzo et al., 2024).

### 2.2. The Impact of Language Arousal on Satisfaction with AI Digital Guides and the Research Hypotheses for Its Mediating Mechanisms

Satisfaction is one of the core indicators for evaluating the effectiveness of museum interpretation (Kuo et al., 2018). In the museum context, visitor satisfaction has multiple sources: it stems not only from the degree to which the interpretive function is fulfilled (Nielsen, 2017) but also from the adequate satisfaction of emotional needs (De Rojas & Camarero, 2008). Research shows that satisfaction is influenced by multiple dimensions, including cognitive fluency, emotional value (particularly the pleasure derived from language appeal) (De Rojas & Camarero, 2008), and the sense of accomplishment from knowledge acquisition (W. Wang et al., 2017). Language arousal, as a key variable affecting the intensity of emotional expression and cognitive stimulation in AI digital guides (Niikuni et al., 2022), has an important impact on satisfaction. Moderate language arousal can significantly enhance the appeal and engagement of the interpretation (D. R. Jones et al., 2018), allowing users to experience a deeper level of emotional satisfaction beyond information reception, thereby directly increasing satisfaction levels. Conversely, language arousal that is too low can make the interpretation seem bland and lack necessary emotional connection, while excessively high arousal may cause irritability, discomfort, or even distrust, ultimately impairing the overall emotional experience and reducing satisfaction (Cascio Rizzo et al., 2024). Furthermore, low-arousal language can also help enhance user satisfaction in specific contexts by creating a more comfortable, stress-free interactive environment (B. Wang et al., 2025). Therefore, this study proposes the following hypothesis:

**H1.**  *Language arousal affects visitor satisfaction with museum digital human guides.*

According to Psychological Distance Theory (Liberman & Trope, 2014), language arousal is a core strategy for regulating the psychological distance between visitors and exhibits/guides (Massara & Severino, 2013). Language arousal at an appropriate level that matches audience characteristics can effectively shorten this psychological distance (Yao & Rui, 2024) and reduce cognitive barriers. This shortened psychological distance directly enhances users’ sense of intimacy and perceived ease of use (C. Jones et al., 2017), lays the foundation for improving satisfaction (Zheng et al., 2020), and facilitates information absorption by reducing cognitive defensiveness and discomfort (Weidlich et al., 2024). Appropriate and natural language arousal helps AI guides transcend the “machine” feel, making them appear more human and understanding (Cascio Rizzo et al., 2024). When users feel that the guide can empathize with the exhibit content or the audience’s feelings, it shortens the psychological distance and establishes a more positive service relationship (Massara & Severino, 2013). Therefore, this study proposes the following hypothesis:

**H1a.**  *Psychological distance mediates the relationship between language arousal and visitor satisfaction with digital human guides.*

The degree of language arousal profoundly influences the ability to evoke emotional resonance in viewers (Citron et al., 2019). Moderate arousal (avoiding levels too low to be appealing or too high to seem artificial and exaggerated), combined with contextual narrative and emotional projection, is key to triggering deep emotional connections between visitors and exhibits or historical events, such as a sense of “being there” or awe at natural wonders (Barclay, 2020). Successfully evoked emotional resonance is central to achieving high user satisfaction, as it fosters a profound sense of pleasure and meaning. Barclay (2020) notes that emotions enhance memory and play a key role in museum learning. Appropriate emotional responses can promote information encoding and long-term memory. Therefore, this study proposes the following hypothesis:

**H1b.**  *Emotional resonance mediates the relationship between language arousal and visitor satisfaction with digital human guides.*

Language arousal is a key lever for maintaining user concentration. It attracts and sustains attention through information novelty, rhythmic variation, intonation, and emphasis (B. Wang et al., 2025). Appropriate arousal, matched to content complexity, optimizes the auditory structure of information, enhances cognitive fluency, and reduces user processing load. A smooth, uninterrupted cognitive experience is itself an important component of user satisfaction (Chen et al., 2024). Efficient knowledge acquisition and a smooth experience (Chang & Suh, 2025) together form the value foundation supporting users’ continued use intention. Therefore, this study proposes the following hypothesis:

**H1c.**  *Concentration mediates the relationship between language arousal and visitor satisfaction with digital human guides.*

### 2.3. The Impact of Language Arousal on Emotional Resonance with AI Digital Guides and the Research Hypotheses for Its Mediating Mechanisms

Continued use intention reflects the long-term value of digital human interpretation services (Pan et al., 2020), and this intention is highly dependent on users’ overall perception of the emotional value of museum interpretation services (Xue et al., 2025). Regarding the mechanism of language arousal, moderately high language arousal has been shown to be a key factor in creating unique, pleasant, and memorable experiences (W. Huang et al., 2023). Specifically, appropriate language arousal can make the interpretation process itself lively, interesting, and engaging, allowing users to have positive experiences of finding it “interesting” and “touching” (Feng et al., 2022). This positive emotional memory forms the core driver for users to choose the service again or recommend it to others, and is also an important basis for the formation of social sharing intention (Herhausen et al., 2019). Empirical research shows that bland or unpleasant interpretation experiences are unlikely to inspire repeat use intention (Cascio Rizzo et al., 2024). Furthermore, interpretations with high arousal and emotional appeal are more likely to become “highlights” and memorable moments in the user experience (Aryani et al., 2020), possessing inherent potential for social sharing. Users’ active sharing intention and behavior not only reinforce their own recognition of the service’s value but also effectively attract new users, thereby fostering a healthy ecosystem for continued use. It must be emphasized that interpretations lacking sufficient emotional impact often fail to stimulate users’ desire to share (Menon & Kahn, 2002). Therefore, this study proposes the following hypothesis:

**H2.**  *Language arousal affects visitor intention to continue using museum digital human guides.*

According to Psychological Distance Theory, a shortened psychological distance directly enhances users’ sense of intimacy and perceived ease of use, which are key drivers of continued use intention (J. Liu & Wang, 2024). When users feel that the guide can empathize with the exhibit content or the audience’s feelings, it shortens the psychological distance and establishes a more positive service relationship (Massara & Severino, 2013), thereby enhancing continued use intention. This shortened psychological distance directly enhances users’ sense of intimacy and perceived ease of use (C. Jones et al., 2017), which are key drivers of continued use intention (J. Liu & Wang, 2024). Therefore, this study proposes the following hypothesis:

**H2a.**  *Psychological distance mediates the relationship between language arousal and visitor intention to continue using digital human guides.*

Deep emotional connections and the resulting high satisfaction naturally translate into user attachment to the service, positively influencing their continued use (X. Huang et al., 2025). Successfully evoked emotional resonance is central to achieving high user engagement and can effectively promote users’ continued use intention. Successfully evoked emotional resonance is central to achieving high user satisfaction and engagement, as it fosters a profound sense of pleasure and meaning. Therefore, this study proposes the following hypothesis:

**H2b.**  *Emotional resonance mediates the relationship between language arousal and visitor intention to continue using digital human guides.*

Efficient knowledge acquisition and a smooth experience (Chang & Suh, 2025) together form the value foundation supporting users’ continued use intention. Language arousal enhances users’ continued use intention by maintaining concentration and optimizing the cognitive experience. A smooth, uninterrupted cognitive experience is itself an important component of user satisfaction (Chen et al., 2024), and efficient knowledge acquisition and a smooth experience (Chang & Suh, 2025) together form the value foundation supporting users’ continued use intention. Therefore, this study proposes the following hypothesis:

**H2c.**  *Concentration mediates the relationship between language arousal and visitor intention to continue using digital human guides.*

### 2.4. The Impact of Language Arousal on Knowledge Recall After Using AI Digital Guides and the Research Hypotheses for Its Mediating Mechanisms

Knowledge recall is an important objective indicator for measuring the effectiveness of cultural communication (Korzun et al., 2017) and serves a key functional task in the museum learning context. Existing research confirms that emotion plays an indispensable role in museum learning, particularly in enhancing memory (Barclay, 2020). From a cognitive mechanism perspective, cognitive schema integration theory points out that vivid language expression helps establish stronger knowledge associations in the audience’s mind, thereby significantly improving memory effects (Stasenko et al., 2025). Language arousal directly affects users’ knowledge acquisition efficiency by optimizing attention allocation and enhancing cognitive fluency. However, it is important to note the complex relationship between language arousal and knowledge recall: excessively high arousal may cause users to over-focus on emotional stimuli, thereby interfering with the deep encoding and elaborate processing of core knowledge (Ludwig et al., 2013); this interference can even lead to cognitive rejection, ultimately reducing the memory effect for key information. On the other hand, appropriate use of low-arousal language can reduce the interference of emotional vocabulary, allowing users to focus their cognitive resources more intently on understanding and processing the core content; this state of concentration is conducive to improving the memory and recall of information. Furthermore, inappropriate levels of language arousal may also weaken users’ perception of interpretation efficiency, thereby undermining the instrumental value that supports continued use intention (Yin et al., 2017). Therefore, this study proposes the following hypothesis:

**H3.**  *Language arousal affects visitor knowledge recall of museum digital human guides.*

According to Psychological Distance Theory, a shortened psychological distance promotes information absorption by reducing cognitive defensiveness and discomfort (Weidlich et al., 2024), thereby optimizing knowledge recall effects. When digital human guides use appropriate language arousal to shorten the psychological distance, it can effectively enhance the audience’s information reception and memory effects. A shortened psychological distance lays the foundation for promoting information absorption by reducing cognitive defensiveness and discomfort (Weidlich et al., 2024). Therefore, this study proposes the following hypothesis:

**H3a.**  *Psychological distance mediates the relationship between language arousal and visitor knowledge recall.*

The emotional experience accompanied by appropriately arousing language acts as a powerful “memory anchor”, directly optimizing knowledge recall (L. Wang et al., 2023). Successfully evoked emotional resonance can deepen the audience’s memory encoding of exhibit content and improve long-term memory effects. Barclay (2020) notes that emotions enhance memory and play a key role in museum learning. Appropriate emotional responses can promote information encoding and long-term memory. This emotional experience, accompanied by appropriately arousing language, acts as a powerful “memory anchor”, directly optimizing knowledge recall (L. Wang et al., 2023). Therefore, this study proposes the following hypothesis:

**H3b.**  *Emotional resonance mediates the relationship between language arousal and visitor knowledge recall.*

Maintaining good concentration is a direct prerequisite for ensuring effective information reception, deep processing, and successful encoding into long-term memory, and therefore has a decisive impact on knowledge recall (Li & Yang, 2016). Language arousal directly affects the effectiveness of knowledge recall by regulating the user’s level of concentration. Maintaining good concentration is a direct prerequisite for ensuring effective information reception, deep processing, and successful encoding into long-term memory, and therefore has a decisive impact on knowledge recall (Li & Yang, 2016). Therefore, this study proposes the following hypothesis:

**H3c.**  *Concentration mediates the relationship between language arousal and visitor knowledge recall.*

### 2.5. The Moderated Role of the Popular Science Topic

The richness of museum exhibits determines the significant differences in their explanatory content, which can primarily be categorised into two major categories: social history (e.g., historical events, cultural customs, artistic works) and natural science (e.g., physical principles, biological evolution, geological structures) (Meyer et al., 2023). This difference in subject matter not only manifests itself in the knowledge content but also profoundly influences visitors’ cognitive goals, emotional expectations, and information processing methods (J. Wang et al., 2021), thereby necessitating differentiated language activation strategies for digital guide avatars (Cascio Rizzo et al., 2024).

The core value of social history exhibits lies in their cultural significance, emotional connotations, and storytelling (Bennett, 2020). Visitors to such exhibits aim not only to gain information but also to understand cultural context, experience historical context, engage in deep reflection, and stimulate internal emotional resonance (Baker, 2020). Overly sentimental or dramatic high-arousal language can interfere with visitors’ ability to independently build emotional connections and engage in deep contemplation, and may even be seen as disrespectful or simplistic to the profoundness of history or culture (McWilliam & Bickle, 2017). Meanwhile, low-arousal language, which avoids excessive emotional induction, respects visitors’ autonomy and allows them to internally build emotional connections based on empathy and feelings (Thompson et al., 2022). This directly increases satisfaction (Turan et al., 2025) and the intention to continue using it. Eliminating the distraction of intensely emotional vocabulary allows visitors to focus their cognitive resources more closely on the historical details, cultural context, and symbolic meaning of exhibits (Trémolière et al., 2016). A calm language environment facilitates reflective processing, thereby optimizing the accuracy and relevance of knowledge recall. In short, in social history lectures, low-arousal language, by creating a stress-free, contemplative atmosphere, is more likely to effectively shorten psychological distance, stimulate inherent and deep emotional resonance, and maintain stable focus, thereby positively impacting satisfaction, continued usage intention, and knowledge recall.

The core appeal of natural science exhibits is to understand objective laws, master scientific principles, and understand natural phenomena (Hetland, 2019). However, abstract concepts and complex principles often pose a high cognitive barrier for non-expert audiences (Haruyama et al., 2025) and potentially alienate them. Key audience expectations for such explanations lie not only in accuracy but also in stimulating interest, reducing the level of difficulty, and making abstract concepts vivid and tangible (D. Liu et al., 2023; Pietroni, 2025). More passionate language can effectively awaken audience curiosity and a desire for exploration (Cascio Rizzo et al., 2024), transforming passive reception into active participation, significantly reducing the psychological distance caused by knowledge barriers (Cascio Rizzo et al., 2024), and making audiences perceive science as “interesting” rather than “difficult”. Vivid metaphors, a tone of wonder, and appropriate dramatization can make abstract principles concrete and storytelling (L. Zhang & Bowman, 2022), building a cognitive scaffolding for complex information that is easily digestible. The sense of surprise and excitement created by high-arousal language itself is a powerful memory anchor (B. Wang et al., 2025), significantly improving recall of key scientific concepts and processes. Compared to purely logical presentations that can appear dry, moderately high-arousal language, through its novelty and emotional appeal, is more effective in capturing and maintaining audiences’ interest-driven attention (B. Wang et al., 2025), especially when presented with complex or unfamiliar content. A smooth and engaging explanation enhances satisfaction based on enjoyment and a sense of accomplishment, and strengthens continued usage intention (Cascio Rizzo et al., 2024).

Existing research generally agrees on the importance of content type in communication strategies (Aichouche et al., 2022), but there is still a lack of research that systematically explores how language arousal strategies vary depending on the topic of the explanation (J. Wang et al., 2021) and empirically tests their moderating effects. Therefore, this study explicitly proposes that the topic of the explanation (social history vs. natural science) is a key moderating variable influencing the mediating variables (psychological distance, emotional resonance, and attention). Specifically, we hypothesis that

**H4a.**  *Popular science topics moderate the relationship between language arousal and psychological distance, i.e., low arousal in social history-themed content is more likely to shorten psychological distance; high arousal in natural science-themed content is more likely to shorten psychological distance.*

**H4b.**  *Popular science topics moderate the relationship between language arousal and emotional resonance, i.e., low arousal in social history-themed content is more positively correlated with (intrinsic, deep) emotional resonance; high arousal in natural science-themed content is more positively correlated with (interest-driven) emotional engagement.*

**H4c.**  *Popular science topics moderates the relationship between language arousal and attention, i.e., low arousal in social history-themed content is more conducive to maintaining stable, contemplative attention; high arousal in natural science-themed content is more conducive to capturing and maintaining interest-driven attention, especially when faced with complex content.*

## 3. Research Methods

### 3.1. Participants

Eighty undergraduate or graduate students from a university in southern China voluntarily participated in this study, including 30 males and 50 females, with ages ranging from 17 to 25 years old. Participants were randomly assigned to either the high-arousal language group or the low-arousal language group. Prior to participating in the study, all participants were fully informed about the study’s content and signed a written informed consent form. Upon completion of the study, each participant received 15 CNY as compensation for their time and effort. This study was conducted in strict accordance with academic ethical standards and has been approved by the Academic Ethics Review Committee of Guangdong University of Technology.

### 3.2. Experimental Design, Experimental Materials and Equipment

This study was completed in May 2025 at a university in southern China using a cross-sectional experimental design. The experiment employed a 2 (language arousal: high vs. low) × 2 (science communication theme: sociocultural vs. natural science) mixed-factor design, where language arousal served as the between-subjects variable and science communication theme as the within-subjects variable. The study measured dependent variables including psychological distance, emotional resonance, concentration, satisfaction, intention to continue using, and knowledge recall through questionnaires.

Regarding participant grouping, 80 subjects were randomly assigned to two equally sized experimental groups of 40 participants each: one group received high-arousal language stimuli, while the other received low-arousal language stimuli. All subjects sequentially viewed two science popularization theme videos (sociocultural and natural science). To control for order effects, an intra-subjects balancing design was further implemented within each experimental group: 20 participants in each group viewed the videos in the order “sociocultural → natural science”, while the other 20 viewed them in the order “natural science → sociocultural”.

For experimental material development, the “Magic Word” (Xmov, Shanghai, China) platform was used to create a digitally animated guide with a uniform appearance, ensuring visual consistency. The script content maintained consistent information structure across two science communication themes (Taiwan Museum and Sengebaum Museum), but manipulated arousal levels through linguistic expression: the high-arousal group script employs an expressive style characterized by passion and significant variations in tone, while the low-arousal group script adopts a calm, steady narrative style. This resulted in four sets of video materials, each combining high/low arousal levels with social-cultural/natural science themes. All video content originated from MOOC courses, ensuring controllable and consistent source material.

**Figure 1:**
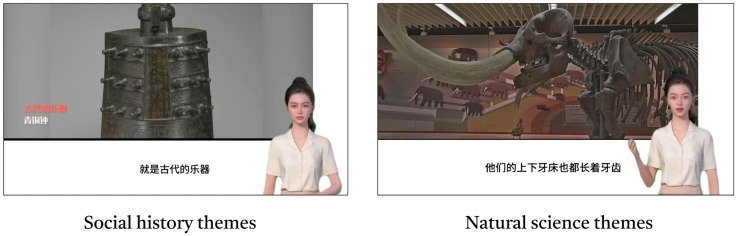
Experimental video format example. Note: The non-English languages shown in the screenshots of the image clock video are Chinese. Since the participants in the experiment were Chinese, Chinese subtitles were added to the digital human’s language.

The prototype of the experimental video is shown in Figure 1. All videos were displayed on a single LCD screen, and a loudspeaker was also installed in the experimental environment. This combination of equipment aimed to replicate the auditory quality and sound effects of a real-life digital human’s explanation as closely as possible, simulating a listening experience close to that of an actual museum tour. To eliminate variables unrelated to the experiment, the subtitle font size and color, the digital human’s position, screen area, body movements, clothing, speaking speed, and timbre remained constant throughout the experiment. All experimental videos were produced using the 3D digital human creation platform “Magic Words” (The official CapCut website “https://www.youyan3d.com/” was visited on 25 March 2025) and the video editing software “Jianying” (The official CapCut website “https://www.capcut.cn/” was visited on 29 March 2025).

### 3.3. Measuring Tools

A questionnaire was developed through a series of steps to measure the relevant research variables. First, the literature on museum experience and language arousal was reviewed, and some questions were modified to suit the current topic of digital human guides in museums. Subsequently, three questionnaire design experts were invited to review and provide suggestions on the questionnaire items. The final questionnaire was refined in detail based on the experts’ suggestions. It consists of three parts: the first part is the demographic attribute section, which collects information on gender, age, and education level; the second part assesses participants’ memory of the digital human’s explanation content, using six questions for each type of explanation. The experts confirmed that the difficulty of the two types of explanations is consistent; the third part evaluates the five scale measurement variables involved in the model. The scale items measure five variables: psychological distance, emotional resonance, concentration, satisfaction, and willingness to continue using. A complete list of scale items and reference sources are provided in Appendix A.

#### 3.3.1. Psychological Distance Scale

Drawing on the perceived usefulness scales of Chen et al. (2024) and Li and Sung (2021), the current study used five items to measure “psychological distance”. Participants were asked to rate their perceived psychological distance from the digital tour guide, for example, “I believe the information provided by the digital tour guide is reliable”. This was measured on a five-point Likert scale, with higher scores indicating closer psychological distance from the digital tour guide, and vice versa. In this study, Cronbach’s alpha coefficient for the scale was 0.914.

#### 3.3.2. Emotional Resonance Scale

Referencing the emotional resonance scale of Escalas and Stern (2003), participants were asked to rate their synchronicity and empathy with the digital figures explaining the corresponding themes of the museum. For example, “The guide’s narrative impressed me with the wisdom of the ancients/the evolution of life”. This scale was scored on a five-point Likert scale, encompassing ten items. In this study, Cronbach’s alpha coefficient was 0.939.

#### 3.3.3. Concentration Scale

Drawing on the focus scales of Li and Yang (2016) and Lu and Yang (2018), participants were asked to rate their own focus during the AI digital human’s explanations, for example, “My attention is not easily distracted when the digital human is explaining the museum”. This scale was scored on a five-point Likert scale, with a total of five items. In this study, the Cronbach’s alpha coefficient for the scale was 0.824.

#### 3.3.4. Satisfaction Scale

Using the satisfaction scale developed by W.-T. Wang et al. (2019), respondents were asked to rate their satisfaction with using AI digital human guides as museum tour guides, for example, “I am very satisfied with the language style of this digital human guide during this type of museum tour”. This scale was scored on a five-point Likert scale with four items. In this study, the Cronbach’s alpha coefficient for the scale was 0.908.

#### 3.3.5. Continuous Use Intention Scale

Referring to the continuance intention scale developed by Xie et al. (2025), participants were asked to rate their willingness to continue using AI digital human guides as museum tour guides, for example, “If possible, I intend to use this digital human guide more often during future museum visits”. This scale was scored on a five-point Likert scale with three items. In this study, the Cronbach’s alpha coefficient for the scale was 0.903.

### 3.4. Experimental Procedures

**Figure 2:**
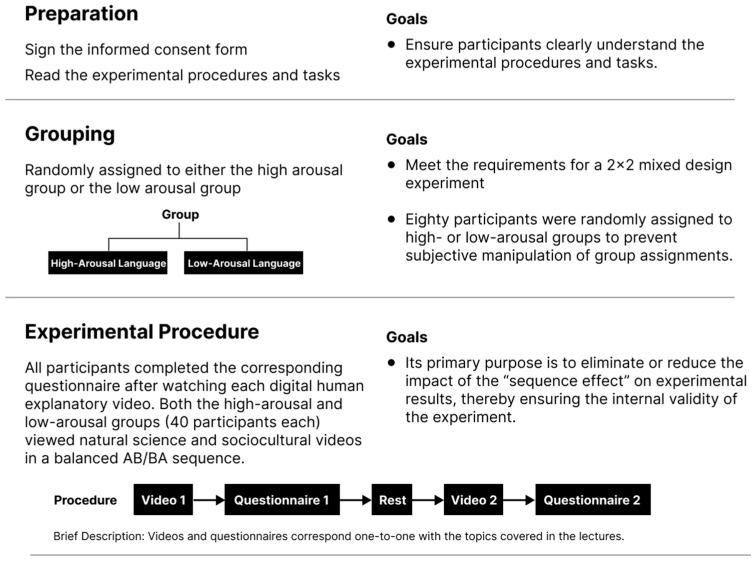
Schematic Diagram of Experimental Design.

The experimental process is as follows: participants are randomly assigned to high-arousal or low-arousal language situation groups. Each group of subjects watches one social culture and one natural science explanation video in the corresponding language version in turn; after watching each video, they immediately fill out the accompanying questionnaire. Figure 2 illustrates the experimental design.

### 3.5. Data Processing Methods

All statistical analyses in this study were conducted using SPSS 27.0. The specific data processing workflow and methods are as follows:

First, repeated-measures analysis of variance (ANOVA) was conducted to examine the main effects and interactions of linguistic arousal (between-subjects variable) and science popularization themes (within-subjects variable) on psychological distance, emotional resonance, attentional focus, satisfaction, intention to continue using, and knowledge recall. This analysis was based on a linear mixed-effects model framework, suitable for handling within-subjects repeated-measures data, and met the sphericity assumption.

Second, to examine the mediating mechanisms of psychological distance, emotional resonance, and attentiveness, the PROCESS macro (version 4.1) developed by Hayes (2015) was employed. Bootstrap sampling (repeated 5000 times) was used to estimate indirect effects and their 95% confidence intervals. Specific analysis strategies included: For moderated mediation paths under science popularization themes, Model 7 was employed to examine differences in the “linguistic arousal → psychological distance → outcome variable” pathway across theme types.

All analyses adopted *p* < 0.05 as the statistical significance threshold, with effect sizes reported as partial η^2^ (ANOVA) and Cohen’s d (*t*-tests).

## 4. Results

### 4.1. Analysis of Differences Between Groups in Behavioral Measurements

The means and standard deviations of the behavioral measures are shown in Table 1.

**Figure 3:**
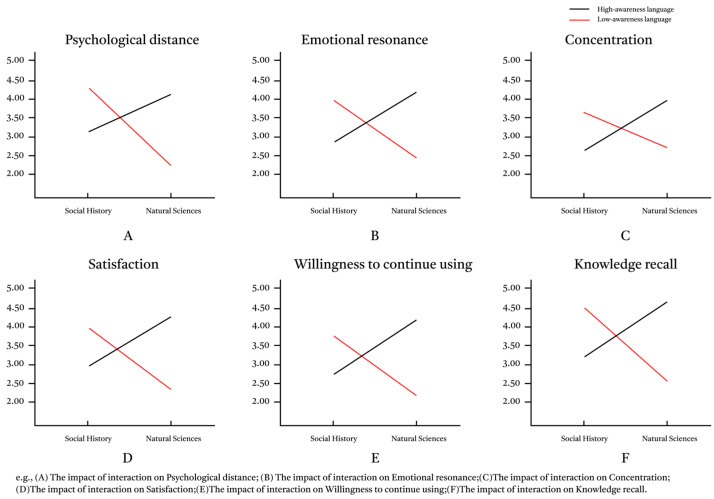
The interactive effect of language arousal and popular science topics.

In order to examine the effects of language arousal (high, low) and subject matter type (social history, natural science) on psychological distance, emotional resonance, concentration, satisfaction, continued use intention, and knowledge recall, a repeated-measures ANOVA was conducted, and the results are shown in Figure 3.

As shown in Table 2, language arousal had a significant main effect on psychological distance, emotional resonance, satisfaction, continued use intention, and knowledge recall, but had no significant main effect on concentration. The main effect of popular science topic was significant only in psychological distance and concentration, but not in the other four dependent variables.

More importantly, there was a highly significant interaction between language arousal and the type of explanation topic, and this interaction reached statistical significance on all six dependent variables (psychological distance, emotional resonance, concentration, satisfaction, continued use intention, and knowledge recall) (all *p* < 0.001). The data is shown in Table 2. Simultaneously, the effect size (partial η^2^) was large, indicating that the combination of the two had a strong dependence on the impact pattern of the results. The results of the simple effect test showed that:

As detailed in Table 3, simple effects tests were conducted to unpack the significant interaction. A consistent pattern emerged under high-arousal language, where natural science themes yielded significantly superior outcomes on all six dependent variables compared to social history themes. Specifically, high arousal reduced psychological distance and boosted emotional resonance, concentration, satisfaction, continued use intention, and knowledge recall for natural science content. This pattern was completely reversed under low-arousal language. In this condition, social history themes led to significantly higher scores across all the same variables, demonstrating the topic-dependent nature of language arousal effects.

### 4.2. Mediation Effect Analysis

#### 4.2.1. The Mediating Effect of Psychological Distance

**Figure 4:**
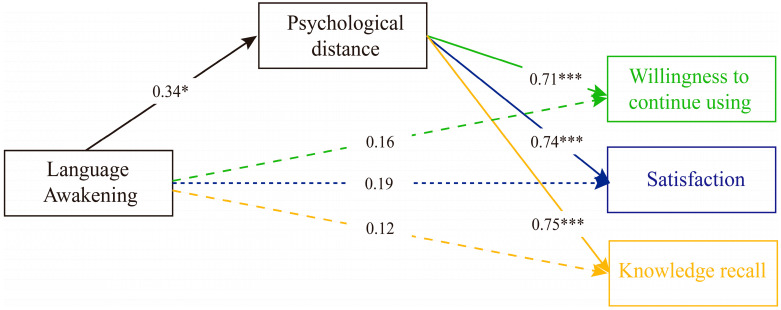
Mediating Role of Psychological Distance in the Effects of Language Arousal on Satisfaction, Continued Use Intention, and Knowledge Recall. Note: * *p* < 0.05, *** *p* < 0.001.

We used process macro model 4 to analyze the mediating effect of psychological distance. As shown in Figure 4, language arousal positively predicted psychological distance (b = 0.34, SE = 0.15, *p* = 0.026), and psychological distance significantly and positively predicted satisfaction with the AI digital human tour guide (b = 0.74, SE = 0.07, *p* < 0.001). The indirect effect of psychological distance was 0.25, with a 95% confidence interval of [0.037, 0.472]. The indirect effect accounted for 50.7% of the total effect (b = 0.44, SE = 0.16, *p* = 0.008).

Secondly, language arousal positively predicted psychological distance (b = 0.35, SE = 0.15, *p* = 0.020), and psychological distance significantly and positively predicted continued use intention of the AI digital human tour guide (b = 0.71, SE = 0.07, *p* < 0.001). The indirect effect of psychological distance was 0.25, with a 95% confidence interval of [0.037, 0.458]. The indirect effect accounted for 61.2% of the total effect (b = 0.41, SE = 0.16, *p* = 0.013).

Finally, language arousal positively predicted psychological distance (b = 0.34, SE = 0.15, *p* = 0.026), and psychological distance significantly and positively predicted the AI digital human’s knowledge recall (b = 0.75, SE = 0.10, *p* < 0.001). The indirect effect of psychological distance was 0.25, with a 95% confidence interval of [0.031, 0.485]. The total effect was not significant (b = 0.38, SE = 0.22, *p* = 0.084).

#### 4.2.2. The Mediating Effect of Emotional Resonance

Analysis of the mediating effect of language arousal on satisfaction, continued use intention, and knowledge recall through emotional resonance revealed that language arousal had no significant effect on emotional resonance (b values were 0.21, 0.22, and 0.21, respectively, with SE values around 0.15, and *p* values > 0.05). However, emotional resonance significantly and positively predicted all three dependent variables (satisfaction: b = 0.81, SE = 0.06, *p* < 0.001; continued use intention: b = 0.73, SE = 0.07, *p* < 0.001; knowledge recall: b = 0.63, SE = 0.10, *p* < 0.001). Further testing revealed that the indirect effect of emotional resonance was not significant in any of the three pathways (indirect effect values were 0.17, 0.16, and 0.13, respectively, with 95% confidence intervals containing 0). In terms of the total effect, language arousal had a significant impact on satisfaction (b = 0.44, SE = 0.17, *p* = 0.008) and continued use intention (b = 0.41, SE = 0.16, *p* = 0.013), but had no significant impact on knowledge recall (b = 0.38, SE = 0.22, *p* = 0.084).

#### 4.2.3. The Mediating Effect of Concentration

The study examined the mediating role of attention in the relationships between language arousal and user satisfaction, continued use intention, and knowledge recall. The results showed that while language arousal had no significant direct effect on attention (b values of 0.08, 0.09, and 0.08, respectively, with SE values ranging from 0.13 to 0.14, and *p* values > 0.05), attention significantly and positively predicted all three dependent variables (satisfaction: b = 0.78, SE = 0.08, *p* < 0.001; continued use intention: b = 0.78, SE = 0.08, *p* < 0.001; and knowledge recall: b = 0.72, SE = 0.12, *p* < 0.001). The indirect effect of attention was not significant in any of the three pathways (indirect effect values of 0.0605, 0.07, and 0.06, respectively, with 95% confidence intervals containing 0). In terms of the total effect, language arousal had a significant effect on satisfaction (b = 0.44, SE = 0.17, *p* = 0.008) and intention to continue using (b = 0.41, SE = 0.16, *p* = 0.013), but had no significant effect on knowledge recall (b = 0.38, SE = 0.22, *p* = 0.084). In terms of the direct effect, after controlling for concentration, language arousal had a significant direct effect on satisfaction (b = 0.38, SE = 0.13, *p* = 0.004) and intention to continue using (b = 0.34, SE = 0.13, *p* = 0.009), but had no significant direct effect on knowledge recall (b = 0.32, SE = 0.195, *p* = 0.103).

### 4.3. Moderated Mediation Effect

#### 4.3.1. Moderated Mediation Effect of Language Arousal → Psychological Distance → Satisfaction

As shown in Table 4, we used the moderated mediation model (Model 7) proposed by Hayes (2015) to test the hypotheses. The direct effects of language arousal on satisfaction and continued use intention were not significant. Psychological distance had a significant positive predictive effect on both satisfaction and continued use intention. Additionally, popular science topics moderated the associations between effect of language arousal on psychological distance, with a significant moderating effect. When the explanation topic was used as the dependent variable, there was a significant main effect of psychological distance on both satisfaction and continued use intention. The key finding was that the topic of instruction significantly moderated the effect of language arousal on psychological distance. All predictor variables collectively explained 47.2% (R^2^ = 0.472) and 44.7% (R^2^ = 0.447) of the variance in the dependent variables satisfaction and continued use intention.

**Figure 5:**
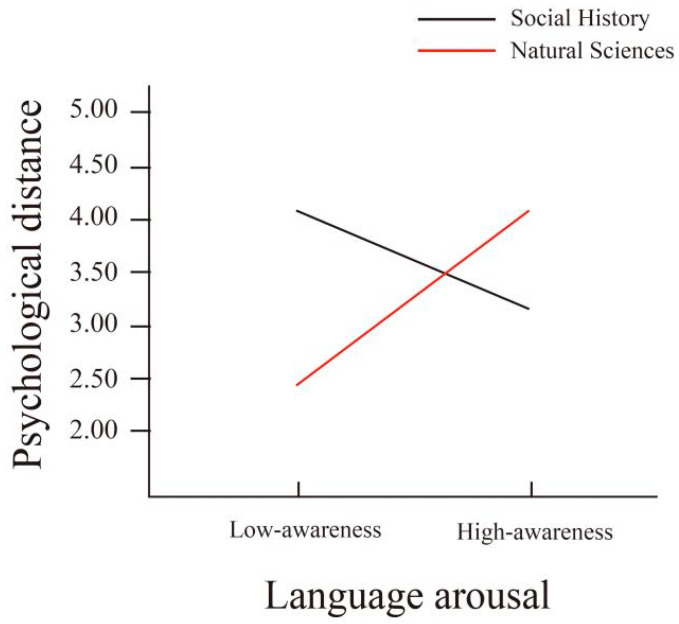
Simple slope plot of the moderating effect of subject matter.

The moderating mechanism of the popular science topic was revealed through a simple slope test. As shown in Figure 5, under the natural science subject matter, language arousal had a significant negative impact on psychological distance (b = −0.97, *t* = 6.51, *p* < 0.001); under the popular science topics, language arousal had a significant positive impact on psychological distance (b = 1.64, *t* = −11.07, *p* < 0.001). Finally, the conditional indirect effect was calculated. The results showed that the indirect effect of psychological distance under the sociocultural subject matter (indirect effect = −1.218, SE = 0.14, 95% CI = [−1.497, −0.944]) was opposite to that under the natural science subject matter (indirect effect = 0.712, SE = 0.12, 95% CI = [0.492, 0.956]). The moderated mediation index was significant (b = 1.922, SE = 0.206, 95% CI [1.522, 2.340]), confirming that the subject matter of the explanation moderated the indirect path of “language arousal → psychological distance → satisfaction”.

#### 4.3.2. Moderated Mediation Effect of Language Arousal → Psychological Distance → Continued Use Intention/Knowledge Recall

As shown in Table 5, we used a Model 7 of the PROCESS macro to test the hypotheses. The direct effects of language arousal on continued use intention and knowledge recall were not significant. Psychological distance had a significant positive predictive effect on continued use intention and knowledge recall. When the explanation topic was used as the dependent variable, psychological distance had a significant main effect on continued use intention and knowledge recall. The key finding was that the topic of instruction significantly moderated the effect of language arousal on psychological distance. All predictors collectively explained 44.7% and 28.7% of the variance in the dependent variables, namely, continued use intention and knowledge recall.

The moderating effect pattern showed significant differences in the impact of language arousal on psychological distance across different explanation topics. The moderated mediation effect of language arousal → psychological distance → continued use intention/knowledge recall was highly similar to that observed when satisfaction was the dependent variable. In the natural sciences topic, language arousal had a significant positive impact on psychological distance (b = 0.97, *t* > 6.35, *p* < 0.001), whereas in the sociocultural topic, language arousal exhibited a significant negative impact on psychological distance (b = −1.64, *t* < −11.07, *p* < 0.001). The indirect effect analysis showed that for continued use intention, the indirect effect under the natural science theme was 0.672, with a Bootstrap 95% CI of [0.437, 0.927], and the indirect effect under the social and cultural theme was −1.164, with a Bootstrap 95% CI of [−1.444, −0.893]. For knowledge recall, the indirect effect under the natural science theme was 0.726, with a Bootstrap 95% CI of [0.449, 1.034], and the indirect effect under the social and cultural theme was −1.235, with a Bootstrap 95% CI of [−1.645, −0.858]. Furthermore, the moderated mediation index for continued use intention was b = 1.835, SE = 0.223, 95% CI [1.408, 2.273], and the moderated mediation index for knowledge recall was b = 1.961, SE = 0.305, 95% CI [1.366, 2.571]. This confirms that the topic of the lecture moderates the indirect path from “language arousal → psychological distance → continued use intention/knowledge recall.”

## 5. Discussion

### 5.1. Relationship Between Language Arousal and AI Digital Guide Satisfaction, Willingness to Continue Using, and Knowledge Recall

This study confirms that language arousal significantly impacts the user experience of digital human docents in museums. However, this effect is subject-dependent, significantly diverging from existing research that suggests a “high arousal advantage” theory. High-arousal language significantly improves satisfaction, continued use intention, and knowledge recall in natural science-themed content. This is because it lowers the threshold for understanding abstract concepts through vivid metaphors and dramatic expressions. This supports Cascio Rizzo et al.’s (2024) assertion that high-arousal language lowers the cognitive threshold through vivid metaphors and aligns with the expectation of language expectancy theory that dynamic content requires high-arousal expression (B. Wang et al., 2025). In contrast, low-arousal language significantly improves satisfaction, continued use intention, and knowledge recall in social history-themed content, consistent with the need for historical narratives to respect audience autonomy (McWilliam & Bickle, 2017). This finding challenges the assumption that high-arousal language is universally effective (B. Wang et al., 2025) and emphasizes that content type determines arousal suitability. This divergence may stem from the specific nature of historical narratives. High-arousal language can be perceived as a simplification of cultural depth (McWilliam & Bickle, 2017), while low-arousal language creates a contemplative space that better meets the audience’s need for independent reflection (Thompson et al., 2022). Methodological differences may also play a role. Previous research has focused on commercial contexts (such as e-commerce live streaming) (W. Huang et al., 2023; B. Wang et al., 2025), while the cultural authority of museums alters audience expectations of appropriate language (Ripatti-Torniainen & Stevanovic, 2023). This study is the first to empirically demonstrate that content type determines arousal adaptability, challenging the assumption that arousal effectiveness is context-neutral.

### 5.2. The Mediating Role of Psychological Distance, Emotional Resonance, and Concentration

Psychological distance is the core mediating variable in the relationship between language arousal and satisfaction, continued use intention, and knowledge recall of AI digital guides, confirming the applicability of psychological distance theory (Liberman & Trope, 2014) to digital human scenarios. This is consistent with Massara and Severino’s (2013) view that language is a core strategy for regulating psychological distance. This empirical research replicates this view in the context of digital humans, demonstrating that language with appropriate arousal can overcome cultural and knowledge barriers. Language arousal indirectly influences all outcome variables by moderating psychological distance (H1a and H3a were supported). In natural sciences, high-arousal language significantly shortens psychological distance, thereby increasing satisfaction and continued use intention. Dynamic language breaks the stereotype that abstract concepts and complex principles in the natural sciences have a high cognitive threshold (Haruyama et al., 2025). It can effectively awaken the audience’s curiosity and desire to explore (Cascio Rizzo et al., 2024), prompting the audience to shift from passively receiving information to actively participating in the cognitive process, thereby significantly shortening the psychological distance caused by knowledge barriers (Cascio Rizzo et al., 2024), transforming science from difficult and obscure to “interesting” in the eyes of the audience. In social history, low-arousal language shortens distance and indirectly increases satisfaction and continued use intention. When strong emotionally charged words are removed from the explanation, the audience can focus more cognitive resources on the historical details, cultural background, and symbolic meaning of the exhibits (Trémolière et al., 2016). The atmosphere created by this calm language is conducive to the audience’s reflective processing of information, and it meets the audience’s psychological expectations of a profound history.

Furthermore, the mediating effects of emotional resonance and concentration were not established (H1b and H3c were not supported): the direct effects of arousal on either were not significant. This finding contradicts Citron et al. (2019), who demonstrated that high-arousal language directly activates the limbic system and triggers emotional resonance, and also diverges from Barclay (2020), who emphasized emotional resonance as a key anchor for museum memory. In-depth analysis suggests that the failure of the emotional resonance mediation pathway may stem from an “emotional authenticity gap” in digital human interactions—while language arousal can convey basic emotional signals, digital humans lack the emotional continuity and contextual adaptability possessed by real guides, making it difficult to establish sustained emotional connections. This may be because emotional resonance relies on genuine human interaction, while current AI-based interactive empathy has limitations. Digital humans lack the emotional authenticity of real people, making it difficult to trigger deep empathy (Li & Sung, 2021). Alternatively, digital humans lack subconscious emotional signals such as micro-expressions and vocal inflection (Tastemirova et al., 2022), making it difficult to elicit profound empathy. This may lead to audience skepticism about the authenticity of “machine emotions” (Li & Sung, 2021), thereby hindering emotional projection. Concentration is more influenced by individual cognitive styles, and audience cognitive styles (such as field dependence/independence) may interfere with the uniform effect of language arousal on concentration (Li & Yang, 2016). Particularly noteworthy is that in the knowledge-authoritative environment of museums, high-arousal language may trigger cognitive vigilance rather than cognitive attraction as seen in commercial contexts. The audience’s expectation of historical authenticity causes overly dramatic expressions to distract from the core content instead. This mechanistic difference explains why high-arousal strategies effective in commercial settings fail in museum environments. This is also inconsistent with the view proposed by Berger et al. (2023) that high-arousal language captures attention through information novelty. This may be because the museum context carries knowledge authority, and excessively high language arousal may lead the audience to perceive the narrative content as lacking authenticity. This differs significantly from the main field of current research—the commercial domain (Cascio Rizzo et al., 2024).

This study confirms that psychological distance is a core mediating variable, while the mediating effects of emotional resonance and concentration are negligible, potentially revealing mechanistic differences between AI interactions and traditional interpersonal communication. In AI communication, psychological distance may be a more robust mediating pathway than emotional arousal. When the communication subject shifts from human to AI, the pathway for affective arousal breaks down, and cognitive distance regulation becomes the core mechanism. This suggests that when designing AI digital human tour guides, prioritizing language strategies to narrow psychological distance is crucial. Long-term research and development could lead to the development of multimodal affective computing models to bridge the AI empathy gap.

### 5.3. The Regulatory Role of the Subject Matter

The subject matter not only moderates the relationship between arousal and psychological distance (H7a holds true), but also reconstructs the complete mediation path from “arousal → psychological distance → communication effect”, providing a contextualized interpretation of language expectancy theory. In natural science-related subjects, high-arousal language shortens psychological distance, stimulates interest-oriented focus, and enhances knowledge recall. This aligns with B. Wang et al. (2025) prediction that high-arousal language is well-suited for dynamic content. In social history-related subjects, low-arousal language shortens psychological distance, promotes endogenous emotional resonance, and enhances memory depth. This supports J. Wang et al. (2021) hypothesis that historical narratives require low-intrusive language. This confirms the necessity of subject-specific language strategies (J. Wang et al., 2021), transcending the existing research’s singular focus on technical parameters.

This study found that subject matter type directly reverses the relationship between arousal and psychological distance, while Palombini (2017) argues that historical themes still require moderate arousal. This difference may stem from the mediation of cultural authority. This experiment used authentic museum content (e.g., historical artifacts from Taiwan), and high-arousal language could easily be interpreted as disrespectful to the culture. Furthermore, it may also be due to the audience’s knowledge level. The Chinese student sample already has a basic understanding of historical exhibits in Taiwanese museums, and low-arousal language facilitates deeper processing (Trémolière et al., 2016). This study is the first to verify that subject matter type is a boundary condition for arousal strategies, providing an operational and adaptive framework for the design of digital humans in museums.

### 5.4. Limitations and Shortcomings

This study has several limitations that should be acknowledged. First, the sample size was limited, consisting exclusively of university students aged 17 to 25. This narrow demographic scope excludes important visitor groups such as children, the elderly, and cross-cultural populations, thereby restricting the generalizability of the findings (Z. Zhang et al., 2025). Second, the ecological validity of the experimental setting was constrained. The use of simulated explanations through video viewing in a laboratory environment diverges from the immersive experience of an actual museum visit. This limitation hinders the full replication of the authentic museum visitor experience and calls for future validation through virtual reality scenarios or in situ museum experiments (Kwon et al., 2024). Third, the study only captured immediate effects, leaving the long-term influence of language strategies on cultural memory unexplored. Finally, the operationalization of language arousal was limited to a high-low dichotomy and did not quantify specific linguistic features—such as emotional word density or rhetorical types. Further refinement in this regard would benefit from the integration of computational linguistics methods.

## 6. Conclusions

This study systematically examines the influence mechanism of AI digital guide language arousal levels on museum visitor experiences from an interdisciplinary perspective of cognitive psychology and communication studies. Findings reveal that the impact of language arousal on visitor satisfaction, continued usage intent, and knowledge recall is highly dependent on the type of science communication theme: natural science content benefits from high-arousal language, which enhances experience effectiveness by stimulating interest and lowering cognitive barriers, while social history content benefits more from low-arousal language, fostering contemplative atmospheres and respecting cultural depth to elicit positive responses. Psychological distance serves as the key mediating pathway through which linguistic arousal influences these outcome variables, while the mediating roles of emotional resonance and attentional focus remain unverified.

Theoretically, this study constructs a “content type–language strategy–psychological distance–communication effect” framework, deepening the application of language expectation theory in the cultural heritage field. It empirically validates for the first time the moderating role of science popularization themes on language arousal strategies, revealing that psychological distance holds greater explanatory power than emotional arousal in AI interactions. Practically, museums and curators are advised to establish theme-sensitive language strategy libraries. AI avatar developers should integrate content adaptive algorithms into speech generation systems, driving the transformation of AI guides from “information tools” to “cultural mediators”. This study has several limitations: the sample was restricted to university students, limiting the generalizability of conclusions; the laboratory setting lacked ecological validity, making it difficult to fully replicate the immersive experience of a real museum; the research measured only immediate effects without tracking long-term impacts; and the language arousal manipulation employed only a high-low dichotomy. Future research should focus on the co-design of multimodal interactions, validate long-term cultural dissemination effects in authentic venues, and expand sample diversity across cultures and age groups to establish more inclusive and practical AI tour design principles. Furthermore, longitudinal experiments across multiple time points can capture the sustained impact of linguistic strategies on visitors’ cultural memory and attitude persistence, thereby reducing interference from random factors.

## Figures and Tables

**Table 1:** Means and standard deviations of behavioral measures.

	High Arousal Group				Low Arousal Group			
	Social History		Natural Sciences		Social History		Natural Sciences	
	M	SD	M	SD	M	SD	M	SD
psychological distance	3.120	0.776	4.040	0.507	4.085	0.477	2.400	0.818
Emotional resonance	2.775	0.769	4.078	0.406	3.980	0.525	2.455	0.761
Concentration	2.515	0.640	3.910	0.512	3.625	0.525	2.645	0.633
Satisfaction	2.913	0.986	4.125	0.494	3.875	0.686	2.275	0.820
Continuous Use Intention	2.624	0.810	4.090	0.565	3.742	0.677	2.175	0.712
Knowledge recall	3.180	1.196	4.650	1.027	4.500	0.961	3.730	1.373

**Table 2:** Results of Repeated-Measures ANOVA (Main and Interaction Effects).

Dependent Variable	Effect	F	*p*	Partial η^2^
Psychological Distance	Language Arousal (A)	13.180	<0.001	0.145
	Topic (T)	10.997	0.001	0.124
	A × T	284.128	<0.001	0.646
Emotional Resonance	Language Arousal (A)	4.768	0.032	0.058
	Topic (T)	2.841	0.094	0.018
	A × T	289.969	<0.001	0.650
Concentration	Language Arousal (A)	0.721	0.398	0.009
	Topic (T)	5.048	0.027	0.061
	A × T	176.588	<0.001	0.531
Satisfaction	Language Arousal (A)	16.736	<0.001	0.177
	Topic (T)	3.421	0.067	0.021
	A × T	199.484	<0.001	0.561
Continued Use Intention	Language Arousal (A)	14.023	<0.001	0.154
	Topic (T)	3.109	0.080	0.020
	A × T	246.927	<0.001	0.614
Knowledge Recall	Language Arousal (A)	5.027	0.028	0.061
	Topic (T)	0.844	0.360	0.005
	A × T	115.202	<0.001	0.425

Note: Nat. A = Language Arousal × T = Topic.

**Table 3:** Results of Simple Effects Analysis (Post hoc *t*-tests).

Arousal Condition	Dependent Variable	Topic		*t*	*p*	95% CI	Cohen’s d
		Mean (SD)					
High Arousal		Nat. Sci.	Soc. His.				
	Psychological Distance	4.04 (0.507)	3.12 (0.776)	5.93	<0.001	[0.64, 2.10]	1.05
	Emotional Resonance	4.08 (0.406)	2.78 (0.769)	13.29	<0.001	[1.19, 1.41]	1.96
	Concentration	3.91 (0.512)	2.52 (0.640)	13.29	<0.001	[1.19, 1.60]	2.35
	Satisfaction	4.13 (0.494)	2.91 (0.986)	9.80	<0.001	[0.91, 1.51]	1.52
	Cont. Use Intention	4.09 (0.565)	2.62 (0.810)	12.32	<0.001	[1.27, 1.67]	1.96
	Knowledge Recall	4.65 (1.027)	3.18 (1.196)	8.72	<0.001	[1.17, 1.77]	1.32
Low Arousal		Nat. Sci.	Soc. His.				
	Psychological Distance	2.40 (0.818)	4.09 (0.477)	10.34	<0.001	[−2.10, −0.64]	1.68
	Emotional Resonance	2.46 (0.761)	3.98 (0.437)	15.59	<0.001	[1.43, 1.63]	2.31
	Concentration	2.65 (0.633)	3.63 (0.525)	9.80	<0.001	[0.78, 1.18]	1.72
	Satisfaction	2.28 (0.820)	3.88 (0.686)	12.34	<0.001	[1.30, 1.90]	2.11
	Cont. Use Intention	2.18 (0.712)	3.74 (0.677)	13.75	<0.001	[1.37, 1.77]	2.31
	Knowledge Recall	2.58 (1.059)	4.50 (0.961)	10.10	<0.001	[1.62, 2.22]	1.83

Note: Nat. Sci. = Natural Science; Soc. His. = Social History. All reported *p*-values are significant after controlling for multiple comparisons.

**Table 4:** Results of the path test for the moderated mediation model (for satisfaction).

Path Relationship	Coefficient	Standard Error	*p*-Value	Outcome
Language Arousal → Satisfaction	0.19	0.13	>0.05	Unsupported
Language Arousal → Continued Use Intention	0.16	0.13	>0.05	Unsupported
Psychological Distance → Satisfaction	0.74	0.07	<0.001	Supported
Psychological Distance → Continued Use Intention	0.71	0.07	<0.001	Supported
Language Arousal × Popular Science Topic → Psychological Distance	2.61	0.21	<0.001	Supported

**Table 5:** The Moderating Effect of Topic on the Mediated Path from Language Arousal Through Psychological Distance to Continued Use Intention and Knowledge Recall.

Predictor	Outcome: Continued Use Intention			Outcome: Knowledge Recall		
	Coefficient	Standard Error	*p*-Value	Coefficient	Standard Error	*p*-Value
Language Arousal	0.16	0.13	>0.05	0.12	0.19	>0.05
Psychological Distance	0.71	0.07	<0.001	0.75	0.1	<0.001
Language Arousal × Topic	2.61	0.21	<0.001	2.61	0.21	<0.001
R^2^	0.447			0.287		

## Data Availability

The original contributions presented in the study are included in the article. Further inquiries can be directed to the corresponding author.

## Appendix A

**Table A1:** The complete list of items for each scale and their reference sources.

Construct	Item	Reference
Psychological distance	I think the information provided by the digital human narrator is reliable	(Chen et al., 2024) and (Li & Sung, 2021)
	I think this digital human narrator is trustworthy	
	I am willing to interact with/respond to this digital human explainer frequently	
	I agree with the professional knowledge and capabilities of this AI digital human tour guide	
	I enjoyed watching the digital human guide’s museum tours.	
Emotional resonance	Through the explanation of the digital human tour guide, I can clearly understand the historical background/scientific significance of the exhibits.	Escalas and Stern (2003)
	The guided tour made me realize the cultural value and natural laws behind the exhibits.	
	I tried to use the information in the tour guide to speculate on the role of the exhibits in the social/ecological system at that time.	
	The tour guide helped me sort out the logical connections between different exhibits (such as timeline and species evolution).	
	I can use the knowledge gained from the guided tour to explain to others the uniqueness of these exhibits.	
	While listening to the tour guide, I felt like I was in the historical site/natural environment.	
	The guide’s narrative shocked me about the wisdom of the ancients and the evolution of life.	
	I felt a similar emotional resonance with the creators of the exhibits (ancient people/nature).	
	At the end of the tour, I was still immersed in the cultural/natural atmosphere conveyed by the exhibition.	
	I felt the human destiny/ecological stories behind the exhibits had an emotional impact on me.	
Concentration	When the digital human explains the museum, I can block out most distractions.	Li and Yang (2016) and Lu and Yang (2018)
	When the digital human explains the museum, I focus on learning the content.	
	As the digital human explains the museum, I am immersed in the current task.	
	I was easily distracted by other things while the digital man was explaining the museum.	
	My attention is not easily distracted when the digital man explains the museum.	
Continuous Use Intention	If possible, I plan to use this digital tour guide when I visit museums in the future.	Xie et al. (2025)
	If possible, I plan to use this digital guide more often when visiting museums in the future.	
	I will recommend this museum digital guide to my friends.	
Satisfaction	In this type of museum tour, I am very satisfied with the language style of the digital tour guide.	W.-T. Wang et al. (2019)
	The digital human guide’s language style met my expectations for this type of museum tour.	
	In this type of museum tour, I was very satisfied with the experience of using the language style of this digital human tour guide.	
	In this type of museum tour, the digital tour guide’s language style was satisfactory in meeting my needs.	
